# Supplementary material for: Iterative improvement in the automatic modular design of robot swarms
Source: PeerJ Comput Sci. 2020 Dec 7;6:e322. doi: 10.7717/peerj-cs.322 (PMC7924708; doi:10.7717/peerj-cs.322)
Supplement: Supplemental Information 3 [file peerj-cs-06-322-s003.zip › argos3/doc/api/standalone/a00649_source.html]

ARGoS: plugins/simulator/visualizations/qt-opengl/qtopengl\_lua\_statetree\_item.h Source File


- Main Page
- Related Pages
- Namespaces
- Classes
- Files

- File List
- File Members

# plugins/simulator/visualizations/qt-opengl/qtopengl\_lua\_statetree\_item.h

Go to the documentation of this file.

```
00001 
00006 #ifndef QTOPENGL_LUA_STATETREE_ITEM_H
00007 #define QTOPENGL_LUA_STATETREE_ITEM_H
00008 
00009 namespace argos {
00010    class CQTOpenGLLuaStateTreeItem;
00011 }
00012 
00013 #include <QList>
00014 #include <QVariant>
00015 
00016 namespace argos {
00017 
00018    class CQTOpenGLLuaStateTreeItem {
00019 
00020    public:
00021 
00022       CQTOpenGLLuaStateTreeItem(CQTOpenGLLuaStateTreeItem* pc_parent = 0);
00023       CQTOpenGLLuaStateTreeItem(QList<QVariant>& list_data,
00024                                 CQTOpenGLLuaStateTreeItem* pc_parent = 0);
00025       ~CQTOpenGLLuaStateTreeItem();
00026 
00027       CQTOpenGLLuaStateTreeItem* GetParent();
00028 
00029       CQTOpenGLLuaStateTreeItem* GetChild(size_t un_idx);
00030 
00031       void AddChild(CQTOpenGLLuaStateTreeItem* pc_child);
00032 
00033       void RemoveChild(CQTOpenGLLuaStateTreeItem* pc_child);
00034 
00035       size_t GetNumChildren() const;
00036 
00037       void SortChildren();
00038 
00039       QVariant GetData(int n_col) const;
00040 
00041       int GetRow();
00042 
00043    private:
00044 
00045       QList<QVariant> m_listData;
00046       CQTOpenGLLuaStateTreeItem* m_pcParent;
00047       QList<CQTOpenGLLuaStateTreeItem*> m_listChildren;
00048 
00049    };
00050 
00051 }
00052 
00053 #endif
```

---

Generated on 10 Jul 2018 for ARGoS by 
 1.6.1
